# Recent Progress and Perspectives on Photocathode Materials for CO_2_ Catalytic Reduction

**DOI:** 10.3390/nano13101683

**Published:** 2023-05-19

**Authors:** Kangli Xu, Qingming Zhang, Xiaoxia Zhou, Min Zhu, Hangrong Chen

**Affiliations:** 1 State Key Laboratory of High-Performance Ceramics and Superfine Microstructure, Shanghai Institute of Ceramics, Chinese Academy of Sciences, Shanghai 200050, China; 2 School of Materials Science and Engineering, University of Shanghai for Science and Technology, Shanghai 200093, China

**Keywords:** CO_2_ reduction, photoelectrochemical, fuels, photocathode materials

## Abstract

The continuous consumption of fossil energy and excessive emissions of carbon dioxide (CO_2_) have caused a serious energy crisis and led to the greenhouse effect. Using natural resources to convert CO_2_ into fuel or high-value chemicals is considered to be an effective solution. Photoelectrochemical (PEC) catalysis utilizes abundant solar energy resources, combined with the advantages of photocatalysis (PC) and electrocatalysis (EC), to achieve efficient CO_2_ conversion. In this review, the basic principles and evaluation criteria, of PEC catalytic reduction to CO_2_ (PEC CO_2_RR), are introduced. Next, the recent research progress on typical kinds of photocathode materials for CO_2_ reduction are reviewed, and the structure–function relationships between material composition/structure and activity/selectivity are discussed. Finally, the possible catalytic mechanisms and the challenges of using PEC to reduce CO_2_ are proposed.

## 1. Introduction

With the increasing demand for energy in modern society, the crisis of fossil fuels (such as oil, natural gas, etc.) and the greenhouse effect have ensued [1,2,3,4,5]. Therefore, the need for the development of clean and renewable energy is urgent. Solar energy, as a kind of renewable, clean energy, has limitless potential [6]. Using solar energy converts carbon dioxide (CO_2_) into fuel or high-value chemicals, such as carbon monoxide (CO), formic acid (HCOOH), methanol (CH_3_OH), ethanol (CH_3_CH_2_OH), acetic acid (CH_3_COOH), and so on, and is considered an effective means of solving the energy crisis and mitigating the greenhouse effect [7,8,9,10,11].

Photoelectrochemical (PEC) characteristics of semiconductor nanoparticles convert and store solar energy in the form of chemical bonds, making them an effective catalyst for the carbon dioxide reduction reaction (CO_2_RR) [12,13]. Compared with photocatalysis (PC) [14,15] or electrocatalysis (EC) [16,17], PEC has obvious advantages in CO_2_RR. For instance, PEC CO_2_RR can not only promote the separation of photo-generated charges by an external voltage, efficiently overcoming the energy barrier and achieving higher solar energy conversion efficiency [18,19], but can also decrease the overpotential under the action of solar energy to save energy. In other words, the synergistic effects of light and the applied bias potential could jointly reduce the CO_2_ activation energy and facilitate the reduction of CO_2_ [20].

Generally, PEC CO_2_RR includes three processes: (i) the generation of electron-hole pairs on the semiconductor under photo-excitation, and the adsorption of CO_2_ on the surface of the catalyst; (ii) the separation and transfer of electron-hole pairs on the semiconductor; (iii) the surface catalytic reaction, including H_2_O oxidation by holes and CO_2_ reduction by electrons [21,22]. Some *p*-type semiconductor materials have been applied for PEC CO_2_RR, such as CuO_2_ [23,24,25], GaP [26,27,28], TiO_2_ [29,30], etc. An ideal *p*-type semiconductor should meet certain conditions for its conduction band (CB) and valence band (VB) positions, such as the energy level of the CB being more negative than that of CO_2_ reduction, and the bandwidth being within the range of light absorption. Although PEC has made significant progress in CO_2_RR, there are still significant challenges ahead, mainly including further improving the current density and Faraday efficiency (FE), overcoming the high redox potential to activate CO_2_ and break the C=O bond [31,32], and inhibiting the hydrogen evolution reaction (HER) competing with CO_2_RR [33].

## 2. Basic Configuration of PEC Devices

PEC devices for CO_2_ reduction are generally divided into two types: single chamber electrolytic cell and H-type electrolytic cell, which are closed containers made of Teflon and quartz glass, respectively.

**Figure 1:**
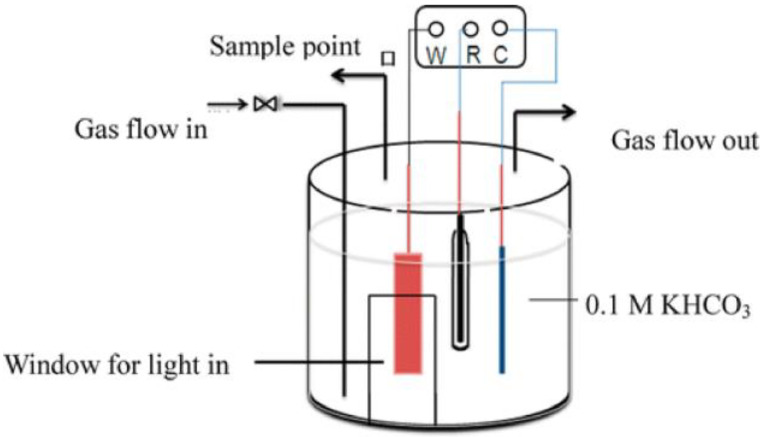
Schematic diagram of the single-chamber reactor. (W, R, and C present the working electrode, reference electrode, and counter electrode, respectively) Adapted with permission from Ref. [34]. Copyright 2014, American Chemical Society.

As shown in Figure 1 [34], in the single-chamber cell, the working electrode, the counter electrode, and the reference electrode are in the same reaction compartment. When CO_2_ is reduced, some of the reduction products (such as HCOOH, CH_3_OH, etc.) can be transferred to the counter electrode and oxidized, resulting in a significant decline in the yield, which is one of the most fatal drawbacks of the single reactor.

**Figure 2:**
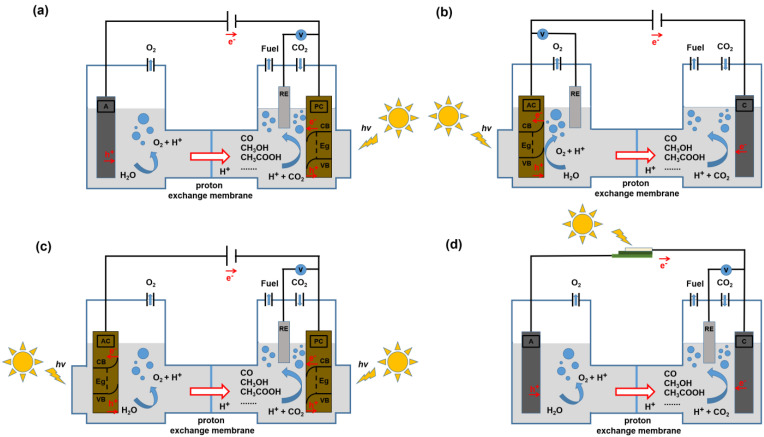
Schematic diagrams of an electrolysis cell for PEC CO_2_RR in a three-electrode system. (**a**) PC, A; (**b**) PA, C; (**c**) PA, PC; (**d**) A, C; and each has a RE.

**Figure 3:**
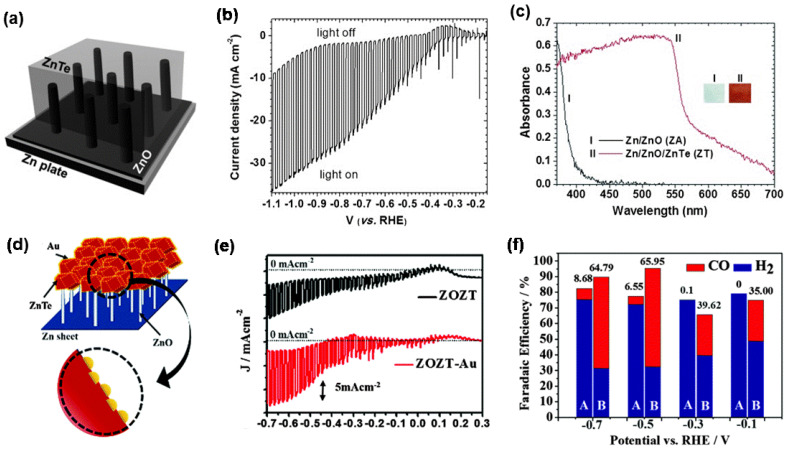
(**a**) Diagram of the Zn/ZnO/ZnTe structure; (**b**) LSV curves of Zn/ZnO/ZnTe with the light on and off; (**c**) UV-Vis absorption spectra of Zn/ZnO and Zn/ZnO/ZnTe. Adapted with permission from Ref. [71]. Copyright 2014, Wiley-VCH. (**d**) Diagram of Au-coupled ZnTe/ZnO/Zn structure; (**e**) LSVs of ZOZT and ZOZT-Au with the light on and off; (**f**) FE for ZOZT (A) and ZOZT-Au (B) (the value above the cylinder indicates the selectivity of CO, concerning H_2_ during H_2_O reduction). Adapted with permission from Ref. [62]. Copyright 2016, Royal Society of Chemistry.

**Figure 4:**
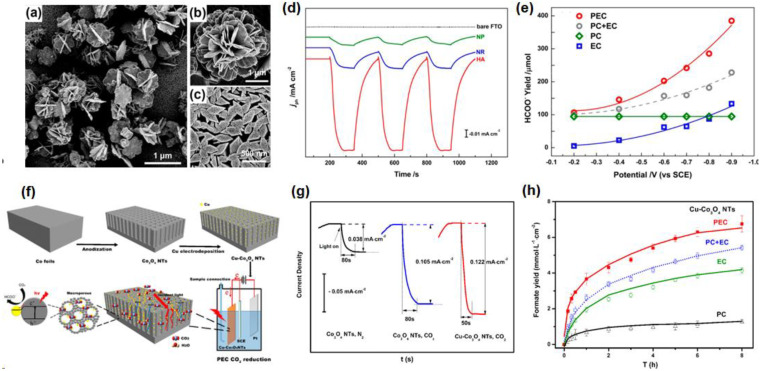
(**a**–**c**) SEM images of HA-Co_3_O_4_ structure; (**d**) *i*-*t* curves of bare FTO, NP-Co_3_O_4_, NR-Co_3_O_4_, and HA-Co_3_O_4_ with the light on/off; (**e**) Formate yields of the sample HA-Co_3_O_4_ in PEC CO_2_RR (red circle), EC (blue square), PC (green rhombus) and PC + EC (gray circle). Adapted with permission from Ref. [74]. Copyright 2013, American Chemical Society. (**f**) Schematic mechanism of the Cu-Co_3_O_4_ NTs fabrication and PEC CO_2_RR; (**g**) *i*-*t* curves of Co_3_O_4_ NTs and Cu-Co_3_O_4_ NTs in N_2_ and CO_2_-saturated electrolyte; (**h**) Formate yields of the sample Cu-Co_3_O_4_ in PEC CO_2_RR (red), EC (green), PC (black) and PC + EC (blue). Adapted with permission from Ref. [75]. Copyright 2015, American Chemical Society.

In the H-type electrolytic cell, the working electrode and the reference electrode are in one compartment, the counter electrode is in another compartment, and the two compartments are separated by a proton membrane. Compared with the single chamber cell, the oxidation reaction occurs in the anode compartment, while the reduction reaction occurs in the cathode compartment, which can effectively prevent the reduction products from being oxidized and can significantly improve the yield of reduction products. However, the transfer of hydrogen protons in the H-type electrolytic cell will be weakened. The three-electrode system of H-type electrolytic cells usually includes the following four types: (1) photocathode (PC, generally *p*-type semiconductor) and dark anode (A, Figure 2a); (2) photoanode (PA, generally *n*-type semiconductor) and dark cathode (C, Figure 2b); (3) PA and PC (Figure 2c); (4) photovoltaic cells and electrodes containing electrochemical catalysts (Figure 2d). Each of the above four electrolytic cells also contains a reference electrode (RE), and the electrolyte can be pure water or a salt solution.

## 3. General Parameters to Evaluate the PEC CO_2_ Reduction

The PEC CO_2_RR efficiency on different catalysts can be evaluated by the following specific standards:

### 3.1. Faraday Efficiency (FE)

FE is one of the important standards to evaluate CO_2_ reduction performance, which can be expressed as the ratio of the actual product yield to the theoretical product yield. FE is a more intuitive way to illustrate the selectivity of a catalyst, and its calculation formula is shown in Equation (1) [35,36,37]:

(1) $$FE_{P}=\frac{I_{P}}{I_{t}}=\frac{n_{P}\;\left(\mathrm{mol}\right)\cdot N\cdot F\;\left(C/\mathrm{mol}\right)}{Q\;\left(C\right)}$$

In Equation (1): *FE_p_* is the Faraday efficiency of *p* (*p* represents the reduction product of CO_2_, such as HCOOH); *I_p_* is the current density of *p*; *I_t_* is the total current density. *n_p_* is the amount of the substance; *N* is the number of electrons that are created for every mole of the corresponding substance. *F* is Faraday constant (*F* = 96,485 C/mol). *Q* is the total amount of charge (*Q* = *I_t_*).

The higher the FE value, the better the selectivity of the catalyst for a specific product. Its value is influenced by multiple factors, such as temperature, electrolyte concentration, applied voltage, solution acidity, and even electrode material purity.

### 3.2. Solar to Fuel Efficiency (STF)

STF is the conversion efficiency of the PEC reduction of CO_2_ system under light irradiation when the action potential between the working electrode and the counter electrode is zero [38,39]. There are two different calculation methods for STF, such as Equations (2) and (3) [35,38,40]:

(2) $$STF=\frac{C_{fuel}\;\left(\mathrm{mmol}/s\right)\cdot △G^{0}\;\left(\mathrm{kJ}/\mathrm{mol}\right)}{P_{solar\;}\left(\mathrm{mW}/{\mathrm{cm}}^{2}\right)\cdot Area\;\left({\mathrm{cm}}^{2}\right)}$$

(3) $$STF=\frac{J_{sc}\;\left(\mathrm{mA}/{\mathrm{cm}}^{2}\right)\cdot △E^{0}\left(V\right)\cdot FE}{P_{solar}\;\left(\mathrm{mW}/{\mathrm{cm}}^{2}\right)}$$

In Equation (2): *C_fuel_* is the amount of fuel produced per unit of time; Δ*G*^0^ is Gibbs free energy that converts CO_2_ into fuel; *P_solar_* is the light power density of incident light and *Area* is the effective area of the working electrode exposed by the incident light.

In Equation (3): *J_sc_* is photocurrent density under short circuit conditions; Δ*E*^0^ is the thermodynamic potential of CO_2_ converted to fuel; *FE* is the Faraday efficiency; *P_solar_* is the light power density of incident light.

### 3.3. Current Density

Current density is the ratio of the tested current to the geometric area of the working electrode, as calculated in Equation (4), which is related to the yield of the product.

(4) $$J_{cd}=\frac{I\;\left(A\right)\cdot 1000}{Area\;\left({\mathrm{cm}}^{2}\right)}$$

In Equation (4): *J_cd_* is current density (mA/cm^2^); *I* (A) represents the total current of the *i*-*t* test, and *Area* is the geometric area of the working electrode.

Based on the total current density and the FE of the corresponding product (such as HCOOH), the partial current density of the corresponding product can be calculated, as shown in Equation (5).

(5) $$J_{pcd}=J_{cd}\cdot FE_{P}$$

In Equation (5): *J_pcd_* is the partial current density of the corresponding product; *FE_p_* is the Faraday efficiency corresponding to the product.

### 3.4. Linear Sweep Voltammetry Curve (LSV)

The onset potential and overpotential of the reaction can be obtained from the LSV curve, which is also an important performance for catalysts.

### 3.5. Incident Photon to Current Efficiency (IPCE)

IPCE is the conversion efficiency of the incident photon into photocurrent under irradiation of incident light with a certain wavelength, which is also called external quantum efficiency [41,42]. IPCE can be calculated by the ratio of the total energy of the converted electrons to the total energy of the incident photons, as shown in Equation (6).

(6) $$IPCE=\frac{1240\cdot J_{sc}\;\left(\frac{\mathrm{mA}}{{\mathrm{cm}}^{2}}\right)}{\lambda \;\left(\mathrm{nm}\right)\cdot P\;\left(\frac{\mathrm{mW}}{{\mathrm{cm}}^{2}}\right)}$$

In Equation (6): *J_sc_* is photocurrent density under short circuit conditions; *λ* is the wavelength of incident light; *P* is the light intensity of incident light at a certain wavelength.

### 3.6. Absorbed Photon-Current Conversion Efficiency (APCE)

APCE is the conversion efficiency of absorbed photons into the photocurrent under light irradiation, which can evaluate the recombination efficiency of photo-generated carriers on the semiconductors as shown in Equation (7) [38,43].

(7) $$APCE=\frac{IPCE\left(\lambda \right)}{A\left(\lambda \right)}$$

In Equation (7): *A*(*λ*) is the absorbance.

### 3.7. Turnover Number (TON) and Turnover Frequency (TOF)

TON and TOF are used to characterize the catalytic activity and stability of the catalyst [44]. TON is the mole number of the conversion substrate divided by the mole number of the catalytic activity center, which can also be expressed as the conversion rate of the active site within a certain period. TOF is the number of conversions of a single active site per unit time. In the photoelectrochemical reduction of CO_2_, TON and TOF are defined as follows (Equations (8) and (9)) [19,24]:

(8) $$TON=\frac{n_{pro}}{n_{cat}}$$

(9) $$TOF=\frac{n_{pro}}{n_{cat}\cdot t}$$

In Equations (8) and (9), *n_pro_* is the mole number of the product; *n_cat_* is the mole number of the catalyst; *t* is reaction time.

### 3.8. Tafel Slope

Tafel slope reflects the relationship between overpotential and current density. The smaller the Tafel slope, the lower the overpotential under the same kinetic current density, indicating better catalytic performance. Moreover, the Tafel slope is also often used to explain reaction mechanisms [45].

### 3.9. Reaction Stability

Stability testing is necessary because most semiconductors are unstable due to photo-corrosion. The amperometric *i*-*t* curve is generally used to describe the stability of the catalyst. In the test, when the current density decreases, the stability attenuates.

## 4. Photocathode Materials for CO_2_RR

Generally, CO_2_RR involves many proton and electron reactions which can produce various reduction products, including CO, CH_4_, HCOOH, CH_3_OH, CH_3_COOH, CH_3_CH_2_OH, etc. To date, metal oxides, metal sulfides, phosphates, etc. have been reported as photoelectrochemical catalysts for PEC CO_2_RR [46,47]. Moreover, a variety of strategies have been proposed to further improve the activity, stability, and selectivity of the catalyst in PEC CO_2_RR, such as the modification of the co-catalyst, and the formation of the protective layer [48]. In this section, we mainly focus on the recent progress of representative photocathode materials to convert CO_2_ into different C products, and their performance enhancement strategies.

### 4.1. Types of Catalysts Commonly Used in PEC CO_2_RR

The development core of PEC technology lies in the development of highly active photoelectric-catalysts, and there are many types of catalysts reported so far for CO_2_ reduction. Among them, metal/oxides (Pt, Au, Fe_2_O_3_, CuFeO_2_, Cu_2_O, TiO_2_, etc.), metal sulfides (CuS, etc.), metal phosphates (GaP, InP, etc.), MOF-based materials (ZIF9-Co_3_O_4_, etc.), and non-metallic carbon materials (g-C_3_N_4_, etc.) have been widely studied. To overcome the overpotential for CO_2_ reduction and drive the reaction with sufficient electrical potential, the conduction band edge of the semiconductor should be much more negative than the potential for CO_2_ reduction. Due to the sufficient negative CB positions, II-VI group materials in the periodic table have received extensive research in the early stages, mainly focusing on the properties of quantum dot materials such as CdTe, ZnTe, CdSe, and CdSeTe. These catalysts typically also have appropriate band gap width and band guide position, which are beneficial for absorbing visible light and reducing CO_2_. In IV group materials, Si has good absorption of ultraviolet light, visible light, and even infrared light. Although the bandgap position of Si satisfies the multi-electron reduction potential of CO_2_RR, it is easily oxidized and the recombination probability of photogenerated carriers is high, making it often used as a substrate. Apart from Si-based catalysts, GaP, InP, Cu_2_O, and CuFeO_2_ are also usually adopted as photocathode substrates.

Based on different semiconductors and reaction systems, the products of PEC CO_2_RR also gradually shift from C_1_ products (CH_4_, CH_3_OH, CO, HCOOH, etc.) to C_2_ products (C_2_H_4_, CH_3_CH_2_OH, CH_3_COOH, etc.) and multicarbon (C_2+_) products (acetone, polyols, etc.). Table 1 summarizes the representative catalysts or active sites for PEC CO_2_ reduction according to the types of CO_2_-reduced products [49]. Similar to the situation in electrocatalytic CO_2_ reduction, Au, Ag-based and Bi, Sn, In-based materials are excellent catalysts for the photocatalytic reduction of CO_2_ to CO and HCOOH in aqueous electrolytes, respectively. The highest FEs for PEC CO and HCOOH production, using these metal-based catalysts, are close to 100%. For example, Fe-based catalysts have been studied extensively for CO_2_RR. The element dopants, together with the local coordination environment of the Fe center, play remarkable roles in affecting the electronic structures and catalytic performances [50]. The reported Fe-based catalysts mainly produce CO with high Fes up to 90% [51,52,53]. However, there are few reports about the selective formation of multicarbon (C_2+_) products over Fe-based catalysts, due to their low FE and low current density. As a comparison, though Cu-based electrocatalysts exhibit unique advantages in electrocatalytic CO_2_ reduction, as they can reduce CO_2_ to various carbon products (CH_3_OH, CH_4_, or C_2+_ chemicals) through multiple electron transfer reactions, it is still a huge challenge to improve the selectivity of a single product, due to the complex and diverse properties. Biological catalysts, including molecular catalysts and enzymes, are also applied in PEC CO_2_ reduction, where the corresponding photocathodes have achieved a record FE of 100% for CO or HCOOH production [54]. In recent years, graphitic carbon nitride (g-C_3_N_4_), as a non-metallic semiconductor material, has shown broad application prospects in CO_2_ reduction research, due to its excellent catalytic performance, which comes from its sufficiently negative CB. Research has shown that pyridine N enriched g-C_3_N_4_, as an active site, can contribute to C-C coupling in PEC CO_2_RR to produce CH_3_CH_2_OH. In addition, vacancy-rich TiO_2_ [55], oxygen-doped CdS, CuFeO_2_, and carbon/Cu_2_O composites are also explored as active sites for PEC CO_2_ reduction to produce CH_3_OH, CH_3_COOH, or CH_3_CH_2_OH, but most of them showed low selectivity for their primary products during the PEC CO_2_ reduction process. To sum up, it remains a significant challenge for electrocatalytic CO_2_RR to produce C_2+_ products efficiently over non-copper catalysts, and there is a lack of photocathodes using metal-based or biological co-catalysts for PEC CO_2_ reduction to produce CH_3_OH, CH_4_, or C_2+_ chemicals with high FEs (>90%).

### 4.2. Strategies for Enhancing the Performance of Photocathodes in PEC CO_2_RR

In recent years, numerous achievements have been made in the research of photocatalytic CO_2_ reduction in photocathode materials. Despite significant progress, there are still many challenges in the efficient reduction of the CO_2_ reaction process. This review is based on the aforementioned PEC CO_2_RR process and briefly elaborates on the basic strategies for enhancing the efficiency of PEC CO_2_RR from three perspectives: light absorption, carrier separation, and surface reaction kinetics.

#### 4.2.1. Improving Light Absorption

The light absorption capacity of semiconductors is closely related to their band structure, therefore, optimizing the band structure of semiconductors is a major strategy for enhancing the absorption performance of photocathodes. In addition, loading light absorbing material onto catalysts is an effective method to enhance their light absorption performance.

Self-doping or doping with other elements can alter the band structure of catalytic materials, thereby improving their light absorption performance. At the same time, the conductivity of the photocathodes is greatly improved, and the current density increases. With regard to PEC systems, there are only a few studies on the doping of photoelectrodes, including Mg-doped CuFeO_2_ [56], B-doped C_3_N_4_ [57], N-doped ZnTe [58], and S, N-co-doped nanoporous carbon [59] photocathodes for PEC reduction of CO_2_.

Plasma metal nanostructures can also improve the efficiency of light energy collection and energy conversion, and have been widely used in semiconductors [60]. Under the action of an incident light wave electric field, the outer free electrons of metal nanoparticles are polarized and move, generating a new electric field that applies a linear internal restoring force within the original system. This type of electronic dipole oscillation, limited to the interior of metal nanoparticles, is known as localized surface plasmon resonance (LSPR). Common precious metals with LSPR effects include Au, Ag, Pt, etc., while non-precious metals mainly include Cu. In catalytic reactions, the abovementioned metal cocatalysts exhibit excellent performance in PEC CO_2_RR.

Incorporating dye molecules and quantum dots into the catalyst can enhance its light absorption ability. Xu et al. [30] utilized multifunctional TiO_2_ thin films as photocathodes to convert CO_2_ into CO, and lower CH_3_CH_2_OH through PEC. The light absorption ability of the photocathodes is improved by combining the dye molecule, Eosin Y Disodium, with TiO_2_, and Pd nanoparticles and amine ligands are introduced to capture protons and CO_2_, respectively.

#### 4.2.2. Enhancing the Separation Efficiency of Photogenerated Carriers

In the process of PEC CO_2_ reduction, a large amount of recombination of photogenerated carriers occurs on the catalyst body and surface, greatly reducing the catalytic reaction efficiency. Therefore, enhancing the separation efficiency of photogenerated carriers is an important means of improving the photoelectrocatalytic efficiency.

Constructing homo/heterojunctions is considered a highly effective strategy for improving the separation of photogenerated electrons and holes, as well as enhancing the selectivity for specific products in PEC CO_2_ reduction [61]. The heterojunction photocathode is usually composed of two or more semiconductors with alternating band structures, such as ZnO/ZnTe [62], CdTe/ZnTe [63], CuO/Cu_2_O [64], and CuFeO_2_/CuO [65], etc.

#### 4.2.3. Accelerating Surface Reaction Kinetics

Interface reaction is a crucial step in the PEC process. As mentioned above, after the generation and separation of photogenerated carriers, they ultimately need to be transferred to the surface of the catalyst to participate in the redox reaction. For the photoelectric reduction of the CO_2_ system, when the photogenerated carriers reach the catalyst surface, electrons participate in the CO_2_RR, and holes participate in the oxidation reaction of H_2_O. Research has shown that strengthening the interface reaction process is crucial for improving the efficiency of PEC CO_2_RR.

Controlling the surface morphology of catalysts is an effective way to accelerate interfacial reactions. By adjusting the surface morphology of the catalyst, it has a high specific surface area and exposes a specific crystal plane, thus promoting the absorption of CO_2_ at its surface active site. In addition, precise control of the morphology of the co catalyst can be an effective method to ensure illumination on the photocathode.

Modifying semiconductor surfaces, using different techniques, is another effective strategy for accelerating interface reactions. The sluggish reaction kinetics of CO_2_ reduction, as a result of the high activation energy or overpotential, largely impedes the reaction process. By using techniques such as chemical etching, electrochemical treatment, or chemical vapor deposition (CVD), catalyst deposition (noble/non-noble metals, MOFs [66], molecular complexes [67], polymers, carbon materials [68]), and passivation with thin protective materials on the surface of photocathodes, the surface of photocathodes can be functionalized, which help to accelerate interface reaction kinetics (accelerate multi-electron reactions, reduce reaction overpotential, and promote the transfer of photogenerated charge carriers to the catalyst surface). In some cases, surface catalysts can also improve the stability of the photoelectrodes by rapidly consuming the surface charge carriers. Furthermore, specific functional catalysts can increase the selectivity for the desired products by manipulating the adsorption strengths of certain reaction intermediates.

### 4.3. Representative Photocathode Materials and Their Performance Enhancement Strategies

#### 4.3.1. Zinc (Zn)-Based Photocathodes

Zinc-based photocathodes, such as ZnTe and ZnO, used in PEC CO_2_RR, mainly produce CO. ZnTe is a p-type semiconductor with a direct bandgap of 2.3 eV, and the conduction band position of ZnTe is relatively negative, which is conducive to transport interface electrons [69,70]. Jang et al. [71] first reported a new Zn/ZnO/ZnTe photocathode for PEC CO_2_RR (Figure 3a). An obvious photoelectric reaction can be observed on the Zn/ZnO/ZnTe photocathode, and under the Hg lamp (with >420 nm cutoff filter), the photocurrent density of the Zn/ZnO/ZnTe photocathode approaches 20 mA·cm^−2^ at −0.7 V vs. the reversible hydrogen electrode (RHE) (Figure 3b). Compared with Zn/ZnO, Zn/ZnO/ZnTe showed significantly improved visible light absorption performance, which is consistent with the result that the light absorption edge can be extended to approximately 570 nm (Figure 3c) [62]. However, due to the competitive reaction of HER, the FE of CO at −0.7 V vs. RHE is as low as 22.9%, and the stability of the Zn/ZnO/ZnTe photocathode also needs to be enhanced. To further improve its performance, Jang et al. coupled Au nanoparticles with ZnTe/ZnO/Zn (ZOZT) to obtain a ZOZT-Au photocathode, as shown in Figure 3d. ZOZT-Au not only further enhanced photocurrent density, but also improved the FE of CO to 58% at −0.7 V vs. RHE, in comparison with ZOZT (Figure 3e–f).

This work offers an effective strategy by using a highly conductive metal substrate and heterojunction structure, which can improve the current density and facilitate the separation of photogenerated carriers. In particular, the deposition of Au nanoparticles could further improve the selectivity for CO and inhibit the competitive H_2_ evolution, as well as promote the electron transfer by forming a Schottky junction with ZnTe at the interface. In addition, Au enhances the stability of the electrode. Coupling metal electrocatalysts with semiconductors or forming heterojunctions between semiconductors can effectively increase current density and promote carrier separation, which is worth learning in the design of optoelectronic materials.

#### 4.3.2. Cobalt (Co)-Based Photocathodes

The most common catalyst in Co-based photocathodes is Co_3_O_4_, which is also a semiconductor material with a band gap of 2.07 eV and a conduction band of −1.05 eV [72,73], and the appropriate band gap width and band guide position are beneficial for absorbing visible light and reducing CO_2_. Zhao et al. carried out a systematic study on Co_3_O_4_ for PEC CO_2_RR. Firstly, the hierarchical porous Co_3_O_4_ (HA-Co_3_O_4_) was designed for PEC CO_2_RR to HCOOH [74], which showed the top micro-flowers and the bottom rhombus structure (Figure 4a–c). This typical hierarchical structure added the active site of photocatalysis and electrochemistry, making the photocurrent density on the HA-Co_3_O_4_ electrode much higher than that on the Co_3_O_4_ rhombus nanorod electrode (NR-Co_3_O_4_) and the Co_3_O_4_ nanoparticle-coated electrode (NP-Co_3_O_4_) (Figure 4d), and the HCOO^−^ yield reached 384.8 ± 7.4 μmol in 8 h, which is higher than the yield of pure EC, PC and EC + PC (Figure 4e). Secondly, the metal Cu nanoparticles were modified on the Co_3_O_4_ nanotube arrays to form a metal/metal oxide composite material (Cu-Co_3_O_4_) for PEC CO_2_RR (Figure 4f) [75]. The Co_3_O_4_ nanotube arrays obtained by in situ growth in cobalt foil, through anodizing technology, can not only reduce the resistance between the substrate and the nanotube arrays, but also provide more active sites to promote light absorption. (Figure 4g). In addition, Cu nanoparticles can also promote the adsorption of reducing intermediates, thereby improving the selectivity of formic acid, resulting in a yield of 6.75 mmol·L^−1^·cm^−2^ of HCOOH in PEC CO_2_RR, with selectivity approaching 100% within 8 h (Figure 4h). The successful application of Cu-Co_3_O_4_ in PEC CO_2_RR once again proves that the coupling between metal and semiconductor is a very desirable design in PEC CO_2_RR.

**Figure 5:**
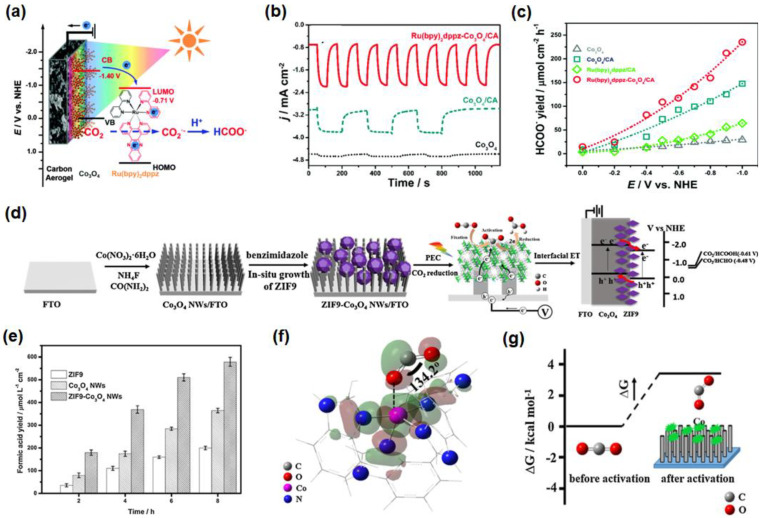
(**a**) Schematic illustration structure of Ru(bpy)_2_dppz-Co_3_O_4_/CA and PEC CO_2_RR; (**b**) *i*-*t* curves of Co_3_O_4_/FTO (dotted line), Co_3_O_4_/CA (dashed line) and Ru(bpy)_2_dppz-Co_3_O_4_/CA (solid line) with the light on and off; (**c**) the yield of HCOO^−^ on Co_3_O_4_/FTO (gray triangle), Ru(bpy)_2_dppz/CA (green rhombus), Co_3_O_4_/CA (cyan square) and Ru(bpy)_2_dppz-Co_3_O_4_/CA (red circle). Adapted with permission from Ref. [72]. Copyright 2016, Royal Society of Chemistry. (**d**) Preparation schematic diagram of ZIF9-Co_3_O_4_ NWs and proposed mechanism of PEC CO_2_RR; (**e**) formate yields on the samples, ZIF9, Co_3_O_4_ NWs, and ZIF9-Co_3_O_4_ NWs electrode in PEC CO_2_RR; (**f**) SOMO orbit of the bent CO_2_-ZIF9 molecule; and (**g**) its corresponding changes in Gibbs energetic Adapted with permission from Ref. [76]. Copyright 2016, Elsevier.

The composite Ru(bpy)_2_dppz-Co_3_O_4_/carbon aerogel (Ru(bpy)_2_dppz-Co_3_O_4_/CA) (Figure 5a), prepared by solvothermal reaction and droplet casting method, also showed superior PEC CO_2_RR performance [72]. The photocurrent response (Figure 5b) and the yield of HCOO^−^ (Figure 5c) on the sample Ru(bpy)_2_dppz-Co_3_O_4_/CA is significantly higher than that of Co_3_O_4_ and Co_3_O_4_/CA, such as the FE of HCOO^−^ is 86% at −0.6V vs. normal hydrogen electrode (NHE). Such a good activity is mainly ascribed to the following aspects: (1) carbon aerogel has rich micropores and a large specific surface area, which can enhance the adsorption of CO_2_; (2) Ru(bpy)_2_dppz can be used as an electron transfer medium and CO_2_ activator, and the synergistic effect between bpy and dppz can also effectively achieve the rapid transfer of electrons. It is believed that modifying the metal/oxide catalysts with organic molecules or linkers, to construct a microenvironment for CO_2_ activation and reduction, will be a new strategy to regulate the photocathode activity or product selectivity.

Additionally, the Co_3_O_4_ nanowires (Co_3_O_4_ NWs), combined with Co-MOF (ZIF9), are conducive to the adsorption, and the capture of CO_2_ has also been applied in PEC CO_2_RR (Figure 5d) [76]. Under light illumination, the yield of formic acid is 578.7 mol·L^−1^·cm^−2^ and the FE of HCOOH is 70.6% in 8 h at −0.9V vs. saturated calomel electrode (SCE) (Figure 5e). DFT theoretical calculation results showed that when CO_2_ was absorbed into the micropores of ZIF9, the structure of linear CO_2_ would change and the bond angle would reduce, due to bending, which could promote the activation of CO_2_ (Figure 5f–g). It has been concluded that the structure of Co_3_O_4_ NWs can enhance light capture, while ZIF9 can improve the adsorption performance of CO_2_. Moreover, the p-p type heterojunction between Co_3_O_4_ NWs and ZIF9 is highly beneficial in promoting the separation of photogenerated electron-hole pairs, showing great potential in CO_2_ reduction. In brief, the research progresses on Co-based photocathodes has paved the way for the development of non-precious metals to achieve PEC reduction of CO_2_.

#### 4.3.3. Silicon (Si)-Based Photocathodes

**Figure 6:**
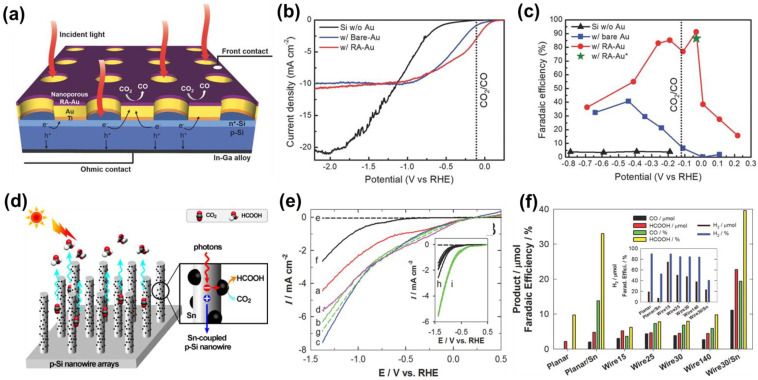
(**a**) Schematic of Au_3_Cu NP/Si NW photoelectrode design for CO_2_ reduction; (**b**) PEC CO_2_RR LSV curves; (**c**) FE of CO; (bare Si—black; Si photoelectrodes of the Au mesh without RA treatment—blue; Si photoelectrode of the Au mesh with RA treatment—red; Green star symbol indicates FE of CO on Si with the Au mesh after the RA treatment). Adapted with permission from Ref. [77]. Copyright 2016, Wiley-VCH. (**d**) Vertically aligned, free-standing p-Si nanowire arrays of varying lengths are grown on p-Si wafers and coupled with Sn nanoparticles for solar CO_2_ conversion; (**e**) LSVs of the irradiated p-Si wire arrays etched for 1, 3, 5, and 10 h (a–d, respectively), planar Si (e—dark; f—irradiated), and p-Si wire array coupled with g—Sn nanoparticles. Inset: 5-times repeated LSVs of the h—irradiated planar, and i—wire electrodes; (**f**) Comparison of the products (CO and formate) and their FE. Inset: the amount of H_2_ produced and FE. Adapted with permission from Ref. [79]. Copyright 2014, Wiley-VCH.

Si, as a potential p-type semiconductor, has a narrow energy bandgap and is widely used in PEC. Moreover, Si is abundant on Earth and has great industrial value [77]. Bradley et al. first reported p-Si photocathode materials in 1982 [78]. In PEC CO_2_RR, p-Si is usually used for substrates and compounded with metal or other semiconductor catalysts. This is because, although Si has strong light capture ability, its CO_2_ reduction activity is weak and it has high selectivity for hydrogen. It has been reported that Si photoelectrodes modified with nanoporous Au thin film mesh, formed by electrochemical reduction of the anodized gold thin film, can be used for PEC CO_2_RR (Figure 6a) [77]. Compared with pure Si photoelectrodes, when the potential is greater than 1.1 V vs. RHE, the photocurrent density of Si photoelectrodes, with nanoporous Au thin film mesh, is reduced, but the initial potential shifts to a positive potential (Figure 6b). Additionally, the FE of CO on Si photoelectrode, with reduced anodic Au (w/RA Au), was 91% at −0.03 V vs. RHE, which is higher than that of pure Si and Si photoelectrode with untreated Au (Figure 6c). The excellent PEC CO_2_RR of the w/RA Au was ascribed to the Au co-catalyst and p-n heterojunctions formed on Si, which jointly facilitated the separation of the photogenerated carrier. 

In addition, Choi et al. [79] reported that Ag-assisted etchants were mixed with HF and H_2_O_2_ to form Si nanowires by etching the Si surface and combined with co-catalyst Sn to convert CO_2_ into HCOOH through PEC CO_2_RR (Figure 6d). Under AM 1.5G light (100 mW·cm^−2^), p-Si wire arrays have higher photocurrent density, more positive onset potential, and better stability. As the etching time increases, the photocurrent first increases and then remains unchanged (Figure 6e). The length of nanowires and the coupling of Sn have an effect on the selectivity of PEC CO_2_RR (Figure 6f). In addition, the FE for HCOOH in the H-type electrolytic cell reached 88.4%, which is higher than that in the single-chamber electrolytic cell. Although p-Si wire arrays can promote photon absorption and carrier separation, due to their high geometric optical path and low reflectivity, the preparation process generally requires highly corrosive hydrofluoric acid or complex etching technologies, making it unsuitable for large-scale operation.

**Figure 7:**
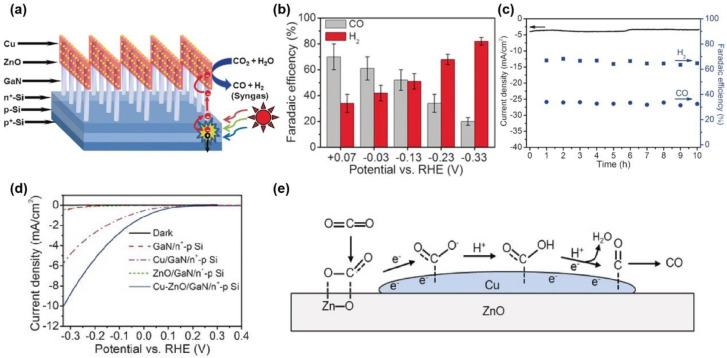
(**a**) Schematic illustration of Cu-ZnO/GaN/n^+^-p Si photoelectrode structure and PEC CO_2_RR; (**b**) relationship between the FE (CO and H_2_) of Cu-ZnO/GaN/ n^+^-p Si photocathode and the potential; (**c**) the stability and FE of Cu-ZnO/GaN/n^+^-p Si photocathode at −0.2 V vs. RHE for CO and H_2_; (**d**) LSV curves of different samples; (**e**) possible reaction mechanism for the PEC CO_2_RR to produce CO on Cu-ZnO cocatalysts. Adapted with permission from Ref. [80]. Copyright 2016, Wiley-VCH.

A p-Si/metal oxide (nitrides)/metal composite was also used for PEC CO_2_RR. Sheng et al. [80] photo-deposited ZnO and Cu on the GaN/n^+^-p Si substrate to convert CO_2_ to syngas using PEC, in which the FE of CO was as high as 70% at −0.2 V vs. RHE in CO_2_-saturated 0.5 M KHCO_3_ solution (pH = 8) (Figure 7a–b). The photocurrent density and FE of CO have no obvious attenuations at −0.2 V vs. RHE under illumination for 10 h (Figure 7c), indicating their high stability, mainly ascribed to the photoabsorption of p-n Si heterojunction, effective electron extraction by GaN, and the fast surface kinetics of the Cu-ZnO co-catalyst. LSV results show that Cu-ZnO/GaN/n^+^-p Si has the highest photocurrent density among all presented samples (Figure 7d), which could be attributed to the ZnO as a cocatalyst to absorb and activate CO_2_ molecules, leading to the bend of the C=O bond and the decrease in CO_2_ chemical stability (Figure 7e). In the Cu-ZnO/GaN/n^+^-p Si photocathode, Si serves as the substrate for light capture, and GaN promotes the separation of photogenerated carriers. Combined with the Cu-ZnO cocatalyst, PEC reduction of CO_2_ can be effectively achieved, which demonstrates the combination of photocatalysts and electrocatalysts, fully achieving the synergistic effects of photo and electricity in material design.

#### 4.3.4. Iron (Fe)-Based Photocathodes

Fe-based photocathode materials mainly include CuFeO_2_ and Fe_2_O_3_. Among them, the bandwidth of CuFeO_2_ is 1.5~1.6 eV, which is beneficial to photon absorption. Furthermore, CuFeO_2_ has the structure of delafossite, and it has been reported that the conductivity of the delafossite structure can be improved by adding trivalent or bivalent metals [56,81]. For example, Gu et al. [56] synthesized Mg-doped CuFeO_2_ by traditional solid-state methods, which showed a high photocurrent density. However, the FE of HCOO^−^ is as low as 10% on the photocathode Mg-doped CuFeO_2_ at −0.9 V vs. SCE, due to the serious hydrogen evolution competitive reaction. Interestingly, CuFeO_2_ and CuO mixed p-type catalysts were prepared to improve the PEC CO_2_RR activity, and the selectivity of HCOO^−^ is close to 90% under simulated light irradiation [65]. More importantly, the activity can last for a week. Moreover, the onset potential of CuFeO_2_/CuO is +0.9 V vs. RHE, which is a very positive potential in PEC CO_2_RR.

**Figure 8:**
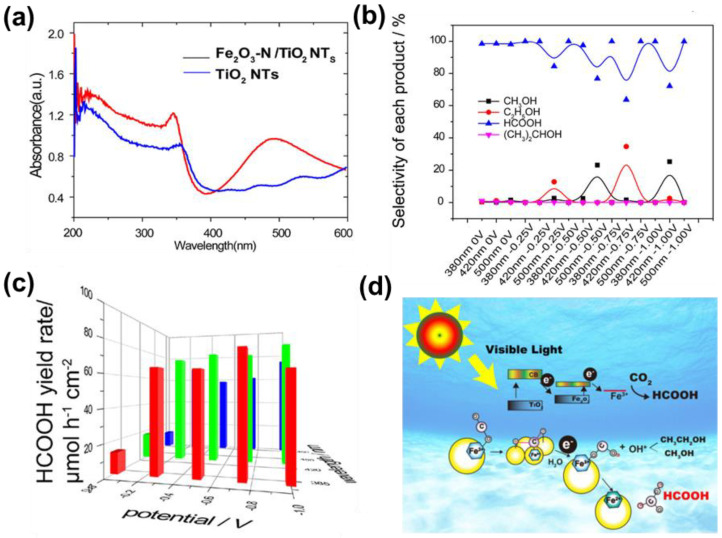
(**a**) UV-DRS of TiO_2_ NTs and Fe_2_O_3_/TiO_2_ NTs; (**b**) selectivity of CO_2_RR products under PC (0 V) and PEC conditions on F_e2_O_3_/TiO_2_ NTs; (**c**) yield rate of HCOOH on Fe_2_O_3_/TiO_2_ NTs; (**d**) proposed reaction pathways for the PEC CO_2_RR on Fe_2_O_3_/TiO_2_ NTs. Adapted with permission from Ref. [83]. Copyright 2018, American Chemical Society.

The bandwidth of transition metal oxide Fe_2_O_3_ is 2.20 eV, therefore it has good visible light absorption performance [82]. Compared with the pure TiO_2_ nanotubes, the TiO_2_ nanotubes modified with Fe_2_O_3_ (Fe_2_O_3_/TiO_2_ NTs) not only expanded the optical absorption region (Figure 8a) but also achieved good PEC CO_2_RR for HCOOH [83]. At an incident wavelength of 500 nm and a bias voltage of −1.0 V vs. RHE, the selectivity and yield of HCOOH can reach 99.89% (Figure 8b) and 74,896.13 nmol·h^−1^·cm^−2^ (Figure 8c), respectively. As shown in Figure 8d, Fe_2_O_3_/TiO_2_ NTs have an embedded heterojunction, and electrons in the VB of TiO_2_ gradually transfer to the CB of Fe_2_O_3_ under incident light irradiation_._ Meanwhile, the Fe_2_O_3_ can absorb CO_2_ to form CO_2_^·−^ (ads) and further obtain [Fe^III^COOH]^2+^ under the effect of electrons and H_2_O. Finally, the HCOOH was formed during the transformation process of O-Fe^III^ and O-Fe^II^. Since Fe-based photocathodes are beneficial for future large-scale applications, the development of efficient non-noble-metal-based co-catalysts should receive much more attention.

#### 4.3.5. Copper (Cu)-Based Photocathodes

Among the non-noble metals, many recent studies on CO_2_ reduction have primarily focused on designing Cu-based materials due to their high abundance, low toxicity, unique catalytic activity, and good stability. There are two main forms of copper oxides, CuO and Cu_2_O. Cu-based photocathodes, especially Cu_2_O, are the most common photoelectrochemical catalysts, due to their good light absorption performance [84,85]. The bandwidth of Cu_2_O is 1.9–2.2 eV, and the CB position is conducive to the reduction of CO_2_ [86]. Additionally, the maximum photocurrent density of Cu_2_O can reach 14.7 mA·cm^−2^ under the illumination of AM 1.5 G light [87]. However, the biggest disadvantage of Cu_2_O is its susceptibility to light corrosion. Therefore, heterojunction and core–shell structure, etc. are often used to effectively avoid photo-corrosion. Additionally, CuO has also a narrow bandwidth of 1.3–1.6 eV, leading to a high absorption coefficient in the solar spectrum [88]. Moreover, Cu-based photocathodes have complex and diverse properties, thus it is challenging to improve the selectivity of a single product.

**Figure 9:**
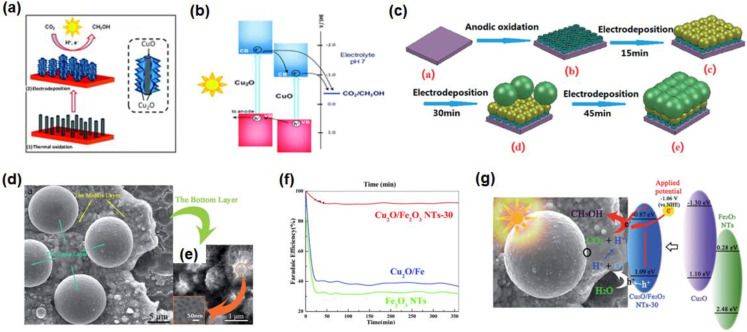
(**a**) Schematic diagram of CuO/Cu_2_O nanorod synthesis and the CO_2_RR; (**b**) energy band diagram and electron transfer route of CuO/Cu_2_O nanorod array for the synthesis of CH_3_OH. Adapted with permission from Ref. [64]. Copyright 2013, Royal Society of Chemistry. (**c**) Growth mechanism of Cu_2_O/Fe_2_O_3_NTs-30 composite; (**d**,**e**) SEM of Cu_2_O/Fe_2_O_3_ NTs-30; (**f**) FE of CH_3_OH on Cu_2_O/Fe_2_O_3_ NTs-30, Cu_2_O/Fe, and Fe_2_O_3_ NTs; (**g**) mechanism of the PEC CO_2_RR on Cu_2_O/Fe_2_O_3_ NTs-30 nanotubes (30: electrodeposition times). Adapted with permission from Ref. [89]. Copyright 2014, Royal Society of Chemistry.

The CuO-Cu_2_O nanorod arrays, as a photocathode, have been prepared on a Cu substrate by a thermal oxidized and electrodeposition process for the reduction of CO_2_ (Figure 9a) [64]. By changing the time of Cu_2_O electrodeposition, the FE of CH_3_OH can reach 94–96% on the sample CuO-Cu_2_O nanorod. Under continuous illumination, the photoexcited electrons can transfer from Cu_2_O to CuO, or directly to CO_2_ adsorbed on the surface of the catalyst, improving the photoelectrochemical performance (Figure 9b). Later, Li et al. prepared Fe_2_O_3_ nanotubes modified with double-layer Cu_2_O spheres (Cu_2_O/Fe_2_O_3_ NTs) by electrodeposition method for PEC CO_2_RR (Figure 9c) [89]. Cu_2_O/Fe_2_O_3_ NTs-30 showed a three-layer structure: the bottom layer was Fe_2_O_3_ nanotubes, the middle layer was Cu_2_O spheres of 200–500 nm, and the top layer was Cu_2_O spheres with a larger diameter (Figure 9d,e). This novel three-layer structure successfully converted CO_2_ to CH_3_OH in 6 h and the FE of CH_3_OH was as high as 93% at −1.3 V vs. SCE, which was higher than Cu_2_O/Fe and Fe_2_O_3_ NTs (Figure 9f). The efficient conversion of CO_2_ into CH_3_OH is mainly because the novel three-layer structure has a conduction band position suitable for CO_2_ reduction and the synergy between PC and EC can promote electron transfer (Figure 9g). Further focus on the effect of Fe:Cu ratio on the product distribution over Cu_2_O/Fe_2_O_3_ will be more helpful in exploring its reaction kinetics and product selectivity.

**Figure 10:**
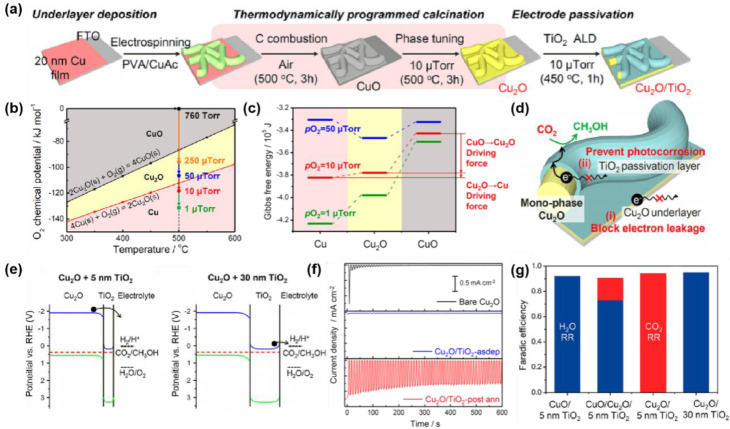
(**a**) The preparation process of hierarchically structured Cu_2_O/TiO_2_ photocathode; (**b**) diagram of the chemical potential of oxygen at different temperatures; (**c**) calculation of the Gibbs free energy of O_2_ at different pressures; (**d**) schematic of the hierarchical structure of Cu_2_O/TiO_2_; (**e**) band diagram of the Cu_2_O/TiO_2_ system with different thickness of the TiO_2_ passivation layer (blue line: CB, green line: VB, red dashed line: forbidden ban); (**f**) the effect of TiO_2_ passivation and the post-annealing process on photostability; (**g**) FE of CO_2_RR for different samples. Adapted with permission from Ref. [90]. Copyright 2018, American Chemical Society.

Recently, Kang et al. [90] prepared a hierarchically structured photocathode material Cu_2_O/TiO_2_ by controlling the synthesis temperature, atmosphere and pressure (Figure 10a). The single-phase Cu_2_O could be obtained by analyzing the chemical potential and accurately calculating the Gibbs free energy of CuO, Cu_2_O, and Cu (Figure 10b,c). In PEC CO_2_RR, TiO_2_ can protect Cu_2_O from corrosion, but the photogenerated electrons, on the CB of Cu_2_O, directly participate in CO_2_RR through the TiO_2_ layer (Figure 10d,e). Interestingly, when the thickness of the TiO_2_ layer is higher than 5 nm, TiO_2_ and Cu_2_O mainly form a p-n heterojunction and the photogenerated electrons on Cu_2_O can transfer to TiO_2_. Therefore, whether TiO_2_ participates in the transfer of electrons mainly depends on its thickness. Through the ingenious design of fewer than 5 nm thick TiO_2_ and pure phase cuprous oxide composite material, the stability and photoactivity have been greatly improved compared with pure Cu_2_O or only deposition TiO_2_ on the pure Cu_2_O but not thermodynamically programmed calcination (Figure 10f). The selectivity of CH_3_OH exceeds 90% on the Cu_2_O/TiO_2_ with 5 nm TiO_2_ layers (Figure 10g), effectively demonstrating that the products based on Cu-based photocathodes achieve high selectivity. Nevertheless, this synthesis method is relatively complex, which is not conducive to large-scale promotion.

#### 4.3.6. g-C_3_N_4_-Based Photoelectrodes

**Figure 11:**
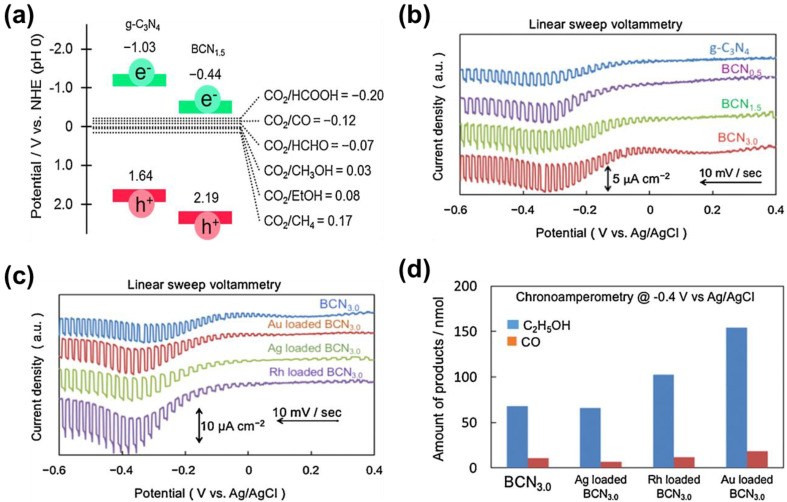
(**a**) Band potential diagram for g-C_3_N_4_ and BCN_1.5_ together with the thermodynamic potentials for CO_2_ reduction to various reduction products vs. NHE at pH 0; (**b**) linear sweep voltammetry of the g-C_3_N_4_ and BCN_x_ electrodes; (**c**) linear sweep voltammetry of the BCN_3.0_ electrodes loaded with or not loaded with co-catalysts; (**d**) product analyses of photoelectrochemical reduction of CO_2_ over co-catalyst loaded BCN_3.0_ electrodes. Adapted with permission from Ref. [57]. Copyright 2016, Elsevier Ltd.

g-C_3_N_4_ is a polymer semiconductor with a bandwidth of 2.7 eV, which has high chemical stability under visible light irradiation, high reduction capacity, and sensitivity to visible light [91,92]. Recently, Nobuhiro Sagara et al. [57] prepared B-doped g-C_3_N_4_ (BCN_x_) for PEC CO_2_RR to CH_3_CH_2_OH. Although g-C_3_N_4_ is an n-type semiconductor, B-doped g-C_3_N_4_ is a proven p-type semiconductor. Compared with the pure g-C_3_N_4_, the potentials of the CB and VB of the sample BCN_x_ are significantly increased, but the potential of the CB is still sufficient to reduce the CO_2_ (Figure 11a). It has been found that the photocurrent response of the sample BCN_x_ could be improved after doping B (Figure 11b). Moreover, the photocurrent response on the different co-catalyst (Au, Ag, Rh)-loaded BCN_3.0_ could be further improved (Figure 11c). Rh or Au-loaded BCN_3.0_ show a higher number of products than BCN_3.0_ (Figure 11d), but the FEs of CH_3_CH_2_OH on Rh-loaded BCN_3.0_ (36%) and Au-loaded BCN_3.0_ (47%) are lower than Ag-loaded, B-doped BCN_3.0_ (73%) and BCN_3.0_ (78%).

**Figure 12:**
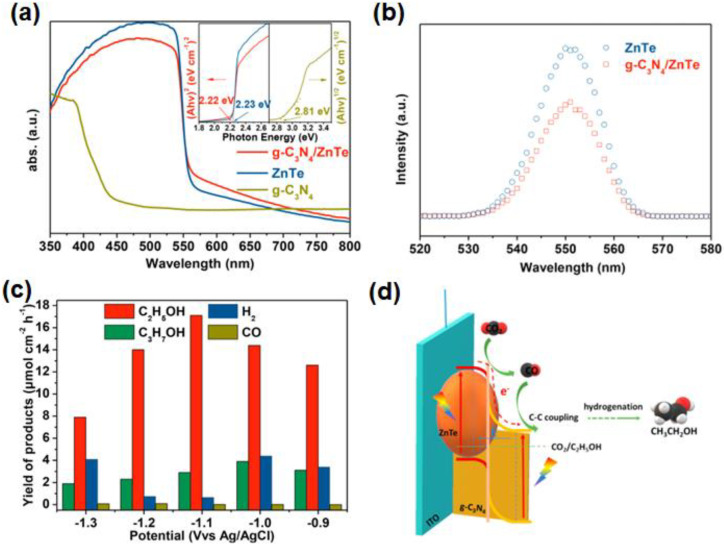
(**a**) UV–vis diffuse reflectance spectra of three photocathodes g-C_3_N_4_/ZnTe, g-C_3_N_4_ and ZnTe; (**b**) PL spectra for g-C_3_N_4_/ZnTe and ZnTe; (**c**) products of g-C_3_N_4_/ZnTe for PEC CO_2_RR at different applied potentials; (**d**) schematic diagram of formed heterojunction and the charge separation process. Adapted with permission from Ref. [93]. Copyright 2019, Elsevier Ltd.

The composite g-C_3_N_4_ and ZnTe, as a photocathode, can form a type II heterojunction and convert CO_2_ to CH_3_CH_2_OH [93]. Through the analysis of the UV-vis absorption spectra, the composite g-C_3_N_4_/ZnTe presents similar visible light adsorption performance and bandwidth to pure ZnTe (Figure 12a). According to PL spectra, it has been found that the peak intensity of the sample g-C_3_N_4_/ZnTe is significantly lower than ZnTe, indicating that the g-C_3_N_4_/ZnTe has a lower electron-hole recombination rate (Figure 12b). The yield of CH_3_CH_2_OH on the heterojunction g-C_3_N_4_/ZnTe is 17.1 μmol cm^−2^·h^−1^ at −1.1 V vs. Ag/AgCl, and the competitive hydrogen evolution is efficiently suppressed (Figure 12c), which is ascribed to the ZnTe with high CO_2_ adsorption capacity, and g-C_3_N_4_ rich in pyridine N contributes to C-C coupling. Moreover, a pipeline mechanism has been proposed to explain the PEC CO_2_RR process (Figure 12d), consisting of three steps: the adsorption and activation of CO_2_, the formation of CO intermediate, and the adsorption of CO intermediate on pyridine N. DFT theoretical calculations indicated that pyridine N sites were very important for the adsorption of intermediates and the formation of ethanol. The results of the studies show that polymer semiconductors, and metal loads used for the construction of heterojunctions, are also effective for PEC CO_2_RR. It is expected that advanced film preparation methods will further improve the film quality of g-C_3_N_4_/ZnTe photocathodes to achieve effective charge separation, which will enhance the activity of PEC CH_3_CH_2_OH production.

#### 4.3.7. Titanium (Ti)-Based Photoelectrodes

**Figure 13:**
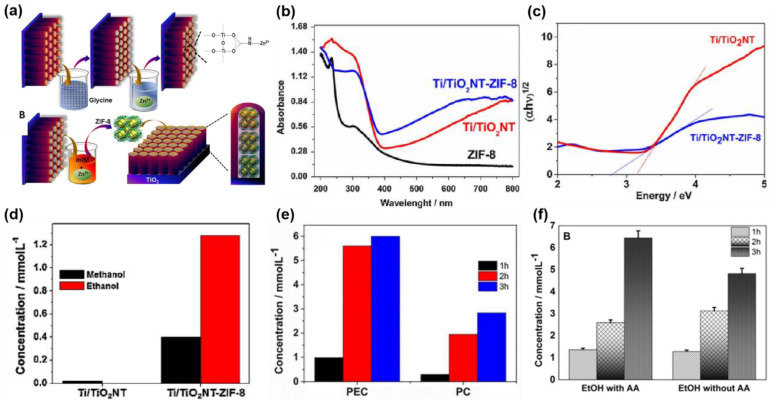
(**a**) Synthetic diagram of Ti/TiO_2_NT-ZIF-8; (**b**) UV–vis absorption spectra of ZIF-8, TiO_2_NT, and TiO_2_NT-ZIF-8; (**c**) Tauc plot of Ti/TiO_2_NT and Ti/TiO_2_NT-ZIF-8; (**d**) concentrations of methanol and ethanol generated in the PEC processes at −0.7 V vs. Ag/AgCl, in 0.1 mol L^−1^ Na_2_SO_4_ (pH 4.5), using the Ti/TiO_2_NT and Ti/TiO_2_NT-ZIF-8 electrodes for 1 h; (**e**) the concentration of ethanol generated by using sample Ti/TiO_2_NT-ZIF-8 in the PEC and PC CO_2_RR. Adapted with permission from Ref. [95]. Copyright 2017, Elsevier B.V. (**f**) The concentration of ethanol on the sample Ti/TiO_2_NT-ZIF-8 with and without ascorbic acid (AA) in the PEC CO_2_RR. Adapted with permission from Ref. [96]. Copyright 2019, Elsevier B.V.

The main structures of TiO_2_ are rutile and anatase, with band gaps of 3.2 eV and 3.0 eV, respectively [94]. TiO_2,_ as the most popular semiconductor, is very stable under ultraviolet light and can be used as a photoanode or a photocathode. However, TiO_2_ shows a weak adsorption performance in the visible range. Cardoso et al. [95] reported that Ti/TiO_2_ nanotubes (TiO_2_NT) modified ZIF-8 as a photocathode for the PEC CO_2_RR (Figure 13a). It has been found that pure ZIF-8 absorbs very little visible light, while the Ti/TiO_2_NT-ZIF-8 shows an enhanced visible light absorption performance and reduced bandwidth, compared with Ti/TiO_2_NT, implying an interaction between nanotubes of Ti/TiO_2_NT and ZIF-8 (Figure 13b,c). In the PEC CO_2_RR, the selectivity to CH_3_CH_2_OH of the sample Ti/TiO_2_NT-ZIF-8 increased significantly, and its conversion rate increased by nearly 430 times in 1 h at −0.7 V vs. Ag/AgCl (Figure 13d). Compared with PC CO_2_RR, the conversion of CO_2_ significantly improved in the PEC CO_2_RR, owing to the synergy of light and electricity (Figure 13e). Moreover, ascorbic acid (AA) as a sacrificial agent was also studied in the PEC CO_2_RR [96]. Sacrificial agents, as donors of electrons, can promote the conversion of CO_2_ and increase CH_3_CH_2_OH conversion, and the FE of CH_3_CH_2_OH reached 86% on the sample Ti/TiO_2_NT and ZIF-8 in the presence of AA (Figure 13f). The reduced N sites of imidazolate groups in ZIF-8 can enhance the adsorption of CO_2_ and form carbamate to promote CO_2_ conversion. Meanwhile, imidazole compounds can act as electron acceptors to enhance light absorption, and thus improve photocurrent response. Such a combination of titania with MOF materials shows great potential in PEC CO_2_RR. Additionally, the results show that adding a sacrificial agent to the reaction is also an effective way to improve the selectivity of the product.

The products and performance of different representative catalysts mentioned above were summarized in Table 2.

## 5. Possible PEC CO_2_RR Mechanisms

CO_2_ has stable chemical properties and low reactivity; thus, high energy is required to realize the conversion of CO_2_**^.^**. Moreover, CO_2_RR is also a complex process involving the coupling of many protons and the electron transfer processes. Using different catalysts and controlling the reaction conditions in the PEC CO_2_RR, different products could be generated. Generally, the conversion of CO_2_ to C_1_ and C_2_ products is easier than other complex hydrocarbon products. Up to now, though the mechanism of CO_2_RR is not particularly clear, many possible reaction pathways have been proposed [21].

Table 3 provides the possible half-reactions of electrochemical CO_2_ reduction and the electrochemical potential of thermodynamically stable CO_2_ molecules into C_1_, C_2_ and C_3_ hydrocarbon molecules. Wherein, the CO_2_ molecule combines with an electron to form anion radical intermediate CO_2_**^·^**^−^, which is generally regarded as the rate-limiting step, since this step needs to overcome a large redox potential, which is much higher than that required by other processes.

**Figure 14:**
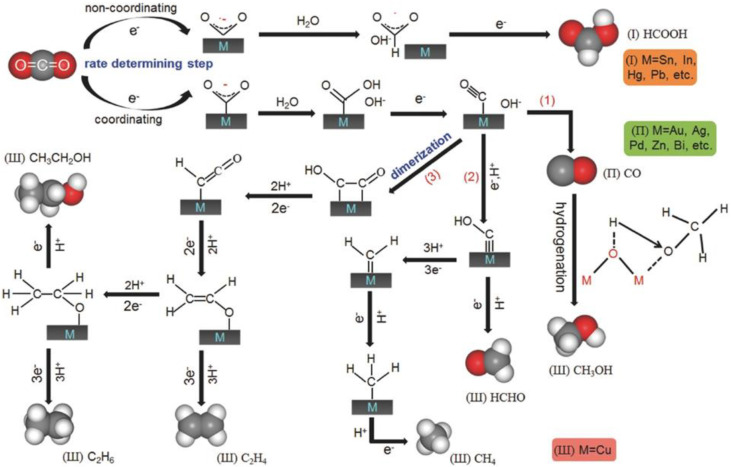
Schematic mechanism of CO_2_ reduction on metal electrodes in aqueous solution. Reprinted with permission from Ref. [97]. Copyright 2018, Wiley.

At present, researchers have explored CO_2_ reduction pathways using elemental catalysts, especially metals, and proposed possible mechanisms of CO_2_ reduction in the aqueous solution, as shown in Figure 14 [97]. In the activation process, the straight CO_2_ molecule can bend as it gains electrons. Additionally, different catalysts have different adsorption strengths for the intermediate and can stabilize different intermediates, thus producing various products, which is essential for the efficient conversion of CO_2_. The main products of CO_2_RR on the transition metals are CO and HCOOH, and the binding energy of the first intermediate and the catalyst influences the final product. When the intermediate CO_2_**^·^**^−^ is weakly adsorbed on the catalyst surface, HCOOH or HCOO^−^ is the main product, and the metal catalysts applied in this reaction are mainly Sn, In, Hg, Pb, etc.

**Figure 15:**
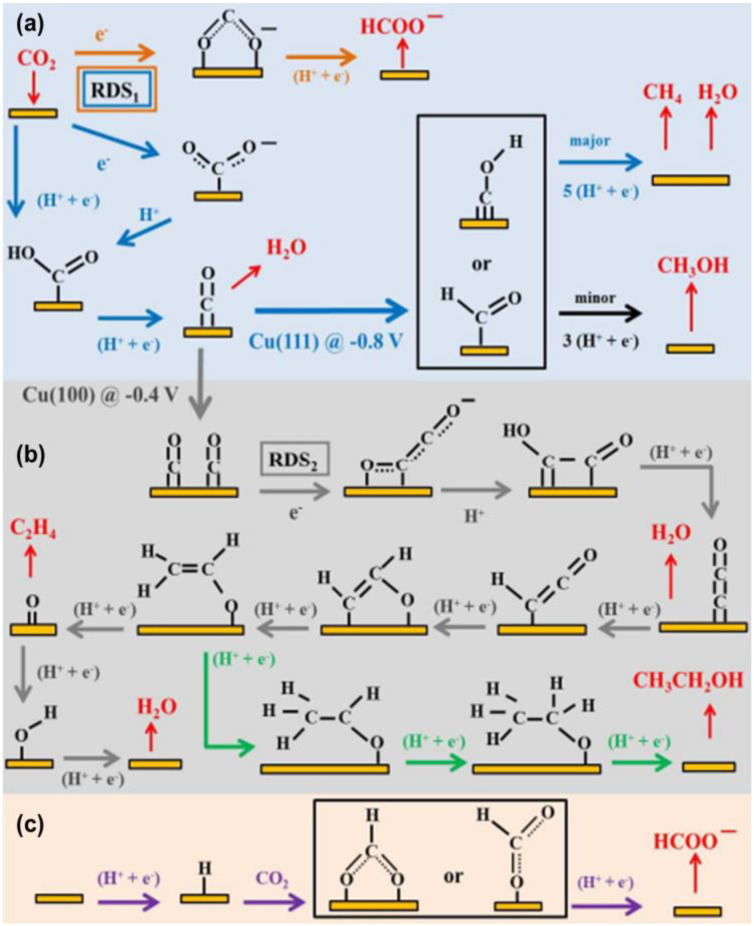
(**a**–**c**) Schematic mechanisms of CO_2_ reduction on Cu electrodes in aqueous solution. Adapted with permission from Ref. [98]. Copyright 2015, American Chemical Society.

Furthermore, some researchers also believe that the intermediate CO_2_**^·^**^−^, that produces HCOOH or HCOO^−^, may also bind to the catalyst by one or two oxygen atoms, but the source of hydrogen for this mechanism is unclear. It is generally believed that hydrogen can include at least two sources: (1) a metallic hydrogen bond; and (2) hydrogen protons in solution (Figure 15a–c) [98]. With regard to the metal catalysts Au, Ag, and Zn, etc., they can be closely combined with CO_2_**^·^**^−^ and *COOH, but it is difficult to combine with the generated *CO intermediate, thus the reduction product is mainly CO. Based on previous research, it is known that Cu is a special metal, which can produce more complex carbon products. Firstly, the *CO intermediate is generated, then the formation of subsequent production products can be roughly divided into three pathways: (1) the combination of electrons and hydrogen protons to form the CO and H_2_O; (2) the conversion of intermediate *CO to *CHO or *COH and the formation of CH_3_OH or CH_4_; (3) dimerizing itself to obtain different products, such as CH_3_CH_2_OH, HCHO, etc. by getting different numbers of electrons and protons (Figure 14 and Figure 15b).

In addition, another mechanism is to avoid the formation of CO_2_**^·^**^−^ intermediate by proton-coupled electron transfer (PCET) [99]. The activation barrier caused by high recombination energy and unstable intermediates is avoided by the transfer of a proton with an electron, but the proton concentration, pH, and temperature and other synthesis conditions have a greater impact on the PECT process [100,101]. 

In summary, there are still many unclear factors and processes in the PEC CO_2_RR, and the research on its catalytic mechanism is still in its infancy. Therefore, we need to further explore the related mechanisms of CO_2_RR, based on the experiment dates, advanced characteristic techniques, and DFT calculations.

## 6. Conclusions, Remarks, and Outlook

In the face of the global energy crisis and increasingly serious environmental issues, the reduction of CO_2_ and the utilization of natural resources (light) have attracted significant attention. The current research, including the design of photocathodes to achieve effective CO_2_ reduction by doping metal and non-metal; construction of heterojunctions such as Z-type heterojunctions, type II heterojunctions and even p-p type heterojunctions; as well as modification of co-catalysts, etc., has made great progress. Nevertheless, there are still great challenges in the practical applications; for example, the problems of low photocurrent density, low FE of reduction products, poor yield of products, and serious photo-corrosion of photocathode materials, etc.

The design of photocathode materials is crucial to the PEC CO_2_RR process. How to improve the light absorption of the designed photocathode material, as well as how to promote the separation of charge carriers and improve the selectivity of the product, etc., will be the emphases of the research. To this end, the following issues should also be taken into consideration:

Firstly, it is crucial to develop a new p-type semiconductor photocathode material with good stability, ultra-visible light adsorption, and CO_2_ conversion performance. Although some p-type semiconductor materials have been developed as photocathodes to achieve the application of PEC CO_2_RR, most of them have serious issues such as photo-corrosion and the complexity of reduction products. Therefore, the developed new p-type photocathode material should have photo corrosion resistance and good selectivity for CO_2_ reduction products. However, the development of new photocathode materials is relatively difficult and requires a long-term process, which requires deeper theoretical foundations and the assistance of DFT calculations.

Secondly, a highly efficient catalyst can be designed by reasonably optimizing the structure and composition, which is currently one of the most universally applicable measures to achieve highly efficient CO_2_ reduction. For example, the combination of photocatalysts with good light absorption and electrocatalysts with super selectivity in CO_2_RR not only preserves the advantages of each component but also regulates the selectivity of the product. More importantly, by loading co-catalysts, visible light can be effectively utilized and photogenerated electrons can be effectively separated. In addition, metals have stronger conductivity, and metal co-catalysts can thus increase current density, making them closer to industrial applications. At the same time, in the design of photocathode materials, combining semiconductor materials with appropriate conduction band positions is also an effective mean of promoting CO_2_ reduction.

Thirdly, improving or optimizing the catalyst preparation process is also an effective way to improve its performance, such as surface coating, surface modification, or a combination of surface technology. Different preparation processes can expose different crystal planes or generate different surface states and configurations, which are conducive to the adsorption of CO_2_ and the desorption of intermediates and products, improving light utilization efficiency, and enhancing the activity and stability of catalytic reactions. Moreover, it is also crucial to control and regulate the contact interface of photocathode materials. Additionally, the contact interface generally serves as a barrier for electron transfer, so precise control of the contact interface can effectively promote the separation of photo-generated charge carriers. For example, more active sites or defects can be exposed at the interface contact with electrolytes, to promote CO_2_ activation.

Last but not least, it is necessary to further strengthen the exploration of CO_2_ reduction mechanisms. A better understanding of the mechanism helps to design the structure of the target catalyst and achieve effective CO_2_ conversion, especially through theoretical calculations, to strengthen the study of the internal relationship between kinetics and thermodynamics.

Overall, PEC CO_2_RR is a promising approach for addressing fossil fuel depletion and mitigating greenhouse effects. Based on the existing technologies, combined with the development of new materials, reasonable design of catalyst structure and components, and optimization or improvement of preparation methods, it is expected to achieve more efficient CO_2_ conversion.

## Figures and Tables

**Table 1:** Summary of the catalysts or active sites for PEC CO_2_ reduction to different products.

Products	Cocatalysts or Active Sites
H_2_	Pt, Ni, Si, etc.
CO	Au, Ag, Pt-TiO_2_, CuAu_3_, AgP_2_, Cu(btc)_3_, ZnO, ZnTe, etc.
HCOOH	Sn, Bi, In, CuS, RuCt, FDH enzyme, CuFeO_2_, Co_3_O_4_, etc.
CH_3_OH	TiO_2_, TiO_2_-Cu^+^, O-doped Cds, etc.
CH_4_	GaN/Cu, GaN/CuFe, Cu-Ag, etc.
CH_3_COOH	CuFeO_2_, etc.
C_2_H_4_ (g)	Cu, Ag/Cu_2_O in AcCN, etc.
CH_3_CH_2_OH	Carbon/Cu_2_O composite, N, O-doped Cds, g-C_3_N_4_, etc.

**Table 2:** Summary of products and PEC CO_2_ reduction performance of different representative photocathodes.

Category	Product	Catalyst	Current Density	FE (%)	Ref.
Zi-based	CO	ZnTe/ZnO (ZOZT)	−7.89 mA·cm^−2^ (at −0.7 V vs. RHE)	7.2	[62]
	CO	ZOZT-Au	−15.97 mA·cm^−2^ (at −0.7 V vs. RHE)	58.0	[62]
Co-based	HCOOH	Cu-Co_3_O_4_ NTs	−0.122 mA·cm^−2^ (at −0.9 V vs. NHE)	-	[75]
	HCOOH	Ru(bpy)_2_dppz-Co_3_O_4_/CA	8.1 mA·cm^−2^ (at −0.6 V vs. NHE)	86	[72]
	HCOOH	ZIF9-Co_3_O_4_	1 mA·cm^−2^ (at −0.9 V vs. SCE)	70.5	[76]
Si-based	CO	Si-w/RA Au	−2.94 mA·cm^−2^ (at −0.6 V vs. RHE)	96	[77]
	HCOOH	*p*-Si/Sn	~−1 mA·cm^−2^ (at −0.6 V vs. RHE)	88.4	[79]
	CO	Cu-ZnO/GaN/*n^+^-p* Si	~−2 mA·cm^−2^ (at +0.07 V vs. RHE)	70	[80]
Fe-based	HCOOH	CuFeO_2_/Mg	−1 mA·cm^−2^ (at −0.9 V vs. SCE)	10	[56]
	HCOOH	CuFeO_2_/CuO	−0.2 mA·cm^−2^ (at +0.9 V vs. RHE)	90	[65]
Cu-based	CH_3_OH	CuO-Cu_2_O nanorod	0.25 mA·cm^−2^ (at −0.2 V vs. SHE)	94–96	[64]
	CH_3_OH	Cu_2_O/Fe_2_O_3_ NTs	~15 μA·cm^−2^ (at −0.6 V vs. SHE)	93	[89]
g-C_3_N_4_-based	CH_3_CH_2_OH	BCN_3.0_	~5 μA·cm^−2^ (at −0.4 V vs. Ag/AgCl)	78	[57]
	CH_3_CH_2_OH	BCN_3.0_/Ag	~10 μA·cm^−2^ (at −0.4 V vs. Ag/AgCl)	73	[57]
Ti-based	CH_3_CH_2_OH	Ti/TiO_2_ NT-ZIF-8	~−1.5 mA·cm^−2^ (at −0.7 V vs. Ag/AgCl)	86	[96]

**Table 3:** The CO_2_ reduction potential in aqueous solutions for the production of different hydrocarbon fuels [97].

Possible Half-Reaction Electrochemical CO_2_ Reduction	Electrode Potentials (V vs. SHE) at pH 7
CO_2_ (g) + e^−^ → *COO^−^	−1.90
CO_2_ (g) + 2H^+^ + 2e^−^ → HCOOH	−0.61
CO_2_ (g) + 2H^+^ + 2e^−^ → CO (g) + H_2_O (l)	−0.53
CO_2_ (g) + 4H^+^ + 2e^−^ → HCHO (l) + H_2_O (l)	−0.48
CO_2_ (g) + 6H^+^ + 6e^−^ → CH_3_OH (l) + H_2_O (l)	−0.38
CO_2_ (g) + 8H^+^ + 8e^−^ → CH_4_ (g) + 2H_2_O (l)	−0.24
CO_2_ (g) + 12H^+^ + 12e^−^ → C_2_H_4_ (g) + 4H_2_O (l)	0.06
2CO_2_ (g) + 12H^+^ + 12e^−^ → CH_3_CH_2_OH (l) + 3H_2_O (l)	0.08
2CO_2_(g) + 14H^+^ + 14e^−^ → CH_3_CH_3_ (g) + 4H_2_O (l)	−0.27

## Abbreviations

PEC	Photoelectrochemical
CO_2_RR	Carbon dioxide reduction reaction
PC	Photocatalysis
EC	lectrochemical
FE	Faraday efficiency
STF	Solar-to-fuel efficiency
IPCE	Incident photon-to-current efficiency
APCE	Absorbed photon-current conversion efficiency
TON	Turnover number
TOF	Turnover frequency
RHE	Reversible hydrogen electrode
NHE	Normal hydrogen electrode
SCE	Saturated calomel electrode
CB	Conduction band
VB	Valence band
AA	Ascorbic acid
PCET	Proton-coupled electron transfer
